# Electrochemical and Optical Experiments and DFT Calculations of 1,4,6,8-Tetrakis((*E*)-2-(thiophen-2-yl)vinyl)azulene †

**DOI:** 10.3390/molecules30183762

**Published:** 2025-09-16

**Authors:** Cornelia Musina (Borsaru), Alina-Giorgiana Brotea, Mihaela Cristea, Gabriela Stanciu, Amalia Stefaniu, Eleonora-Mihaela Ungureanu

**Affiliations:** 1 Faculty of Chemical Engineering and Biotechnologies, National University of Science and Technology Politehnica Bucharest, Gh. Polizu 1–7, Sector 1, 011061 Bucharest, Romania; borsaru_cornelia@yahoo.com; 2 Department of Chemistry and Chemical Engineering, “Ovidius” University Constanta, 900527 Constanta, Romania; alina.brotea@365.univ-ovidius.ro; 3 “C. D. Nenitzescu” Institute of Organic and Supramolecular Chemistry, Romanian Academy, 71141 Bucharest, Romania; mihcris2012@yahoo.ro; 4 National Institute for Chemical—Pharmaceutical Research and Development (ICCF), 031299 Bucharest, Romania; astefaniu@gmail.com; 5 Doctoral School of Chemical Engineering and Biotechnologies, Faculty of Chemical Engineering and Biotechnologies, National University of Science and Technology Politehnica Bucharest, Gh. Polizu 1–7, Sector 1, 011061 Bucharest, Romania

**Keywords:** 1,4,6,8-tetrakis((*E*)-2-(thiophen-2-yl)vinyl)azulene, voltametric methods, UV-Vis, DFT calculation, HMs analysis

## Abstract

Due to its conjugated structure, 1,4,6,8-tetrakis((E)-2-(thiophen-2-yl)vinyl)azulene (**L**) has a high potential for nonlinear optics and coloring. This compound was studied electrochemically using cyclic voltammetry, pulse differential voltammetry and rotating disk voltammetry in organic electrolytes. The main processes occurring during oxidation and reduction scans were highlighted and characterized. Density functional theory (DFT) calculations were conducted to assess the chemical reactivity of this compound. UV-Vis studies of **L** were performed in acetonitrile to establish the optical parameters in this solvent and its complexing power towards heavy metal (HM) ions. Chemically modified electrodes (CMEs) based on **L** were prepared by electrooxidation of **L** in organic electrolytes. To evaluate the electrochemical behavior of the CMEs, they were characterized with a ferrocene redox probe. They were also tested for the analysis of synthetic samples of heavy metal ions (HM): Cd(II), Pb(II), Cu(II) and Hg(II) by anodic stripping. Specific responses were obtained for Pb(II) and Cd(II) ions. The preparation conditions have an influence on the electrochemical responses. This study is relevant for the design and further development of advanced materials based on this azulene for the analysis of HMs in water samples. Electrochemical experiments and DFT calculations recommended **L** as a new ligand for modifying the electrode surface for the analysis of HMs.

## 1. Introduction

**Figure 1:**
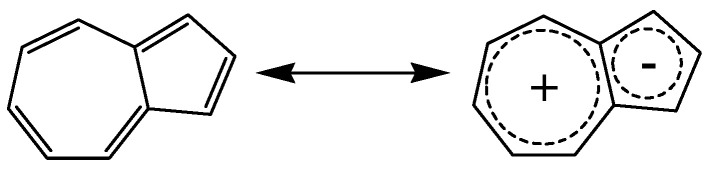
The dipole structure of azulene.

Due to their dipole structure (µ = 1.08 D) [1,2], azulene (Figure 1), a nonalternant bicyclic aromatic hydrocarbon, formed by the condensation of an electron-deficient seven-membered ring (tropylium cation) with an electron-rich five-membered ring (cyclopentadienyl anion), is a structural block for obtaining push–pull systems with advanced optoelectronic properties [3,4,5].

**Figure 2:**
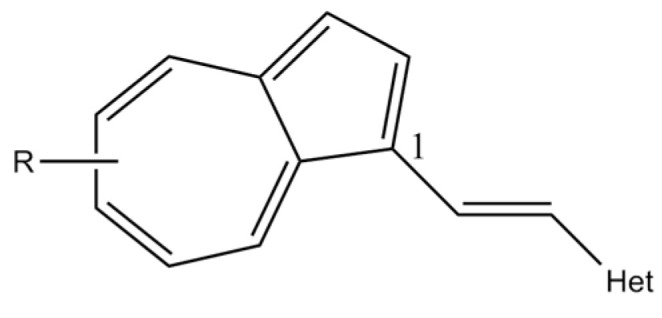
General structure of 1-vinylazulenes with an azulen-1-yl moiety as EDG and a heteroaryl as EWG.

Azulene exhibits good electron-donating capability [6]. A series of push–pull compounds from the class of 1-vinylazulenes with the general structure shown in Figure 2, containing the azulen-1-yl moiety as an electron-donating group (EDG) or linked via a C=C bond to a heteroaryl electron-withdrawing group (EWG), have been reported in the literature [7,8,9,10,11,12,13,14,15,16,17,18,19,20].

Such organic push–pull systems from the 1-vinylazulenes class exhibit high hyperpolarizability, and their investigation is of great research interest in the electrochemistry application field due to their valuable technical properties. According to literature data, these azulene derivatives can be used as nonlinear optical (NLO) systems or as building blocks for other molecules with potential technical applications [3,21,22]. In the field of electrochemistry, 1-vinylazulenes exhibit interesting electrochemical properties, making them suitable for use as modified electrodes with complexing properties [12,23] and as electrochemical sensors for the detection of heavy metal ions in water [24,25,26,27,28,29].

In continuation of our concern regarding obtaining, through electrochemical polymerization, new modified electrodes for the detection of heavy metal ions and contaminants (Cd^2+^, Cu^2+^, Pb^2+^, Hg^2+^) in water at very low concentrations, several azulene monomers based on thiophenvinyl azulene have been tested and reported in our previous papers [25,29].

**Figure 3:**
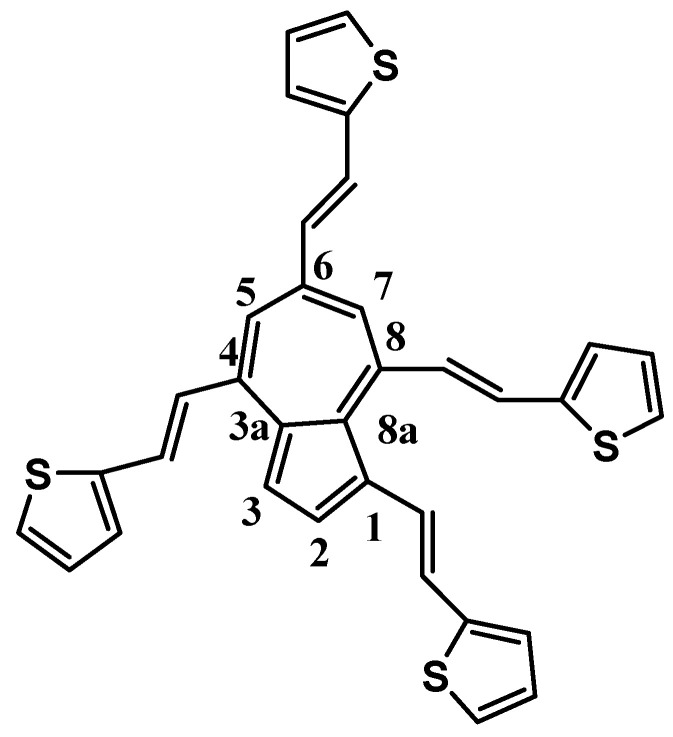
Chemical structure of **L** and numbering of the atoms on azulene.

The title ligand (**L**), namely 1,4,6,8-tetrakis((*E*)-2-(thiophen-2-yl)vinyl)azulene (Figure 3), is an azulene derivative substituted with (thiophen-2-yl)vinyl moieties. Like other azulene derivatives studied in our group, it can have potential nonlinear optical responses and staining properties [30], due to the presence in its molecule of an azulene moiety and of four thienyl groups connected by four conjugated vinyl bonds. Both the thienyl [29] and azulene [11] groups can polymerize, resulting in films.

In the case of electrochemical polymerization, chemically modified electrodes (CMEs) with different applications can be obtained. Since our collective has concerns related to the application of CMEs for heavy metal recognition in waters [25,31], in this work, we performed preliminary electrochemical experiments and DFT calculations for **L**. This new ligand for electrode surface modification was tested for HM ion analysis using CMEs based on this ligand (**L**-CMEs).

Density functional theory (DFT) was performed using the B3LYP/6-31(d,p) method for the equilibrium geometry at the ground state of **L**. Computational properties were calculated using Spartan Software (Wavefunction Inc., Irvine, CA, USA), as in previous studies [25,32].

## 2. Results

1,4,6,8-tetrakis((E)-2-(thiophen-2-yl)vinyl)azulene (**L**) was synthesized and characterized using physicochemical methods to confirm the structure [13]. Detailed results were given in the supplementary material (lines 5–17). This compound was studied using electrochemistry, including cyclic voltammetry (CV), differential pulse voltammetry (DPV), and rotating disk electrode voltammetry (RDE) in organic electrolytes. DFT calculations were also performed on the most stable conformer of **L**, which has an energy minimum, to evaluate its properties responsible for chemical reactivity. Chemically modified electrodes (CMEs) were prepared by electrooxidation of **L**. They were characterized by electrochemistry and were tested for the analysis of heavy metal (HM) ions.

### 2.1. Electrochemistry on ***L***

The ligand **L** was examined by differential pulse voltammetry (DPV), cyclic voltammetry (CV), and rotating disk electrode voltammetry (RDE) in millimolar solutions of **L** in organic electrolytes. Usually, tetrabutylammonium perchlorate (TBAP) dissolved in acetonitrile (ACN) in a concentration of 0.1 M (0.1 M TBAP/ACN) was used. However, in the case of this ligand **L**, which is not very soluble in ACN, dimethylformamide (DMF) was added as a solvent, and the study was performed in mixtures of ACN-DMF 2:1 (0.1 M TBAP in ACN-DMF). These voltametric methods were used to evidence the main oxidation and reduction processes which occur with this molecule when the potential is scanned to anodic or cathodic potentials. Curves were initially recorded separately on the GC electrode (freshly cleaned) in the usual supporting electrolyte for organic substrates (0.1 M TBAP, ACN). The working electrode was polarized starting from the stationary potential to positive potential up to about 3 V, and then the potential returned to its stationary value. After cleaning the electrode, a similar procedure was applied for the potential in the cathodic scan, to about -3 V, and then it was reversed towards positive values to detect all anodic processes corresponding to those generated in the cathodic scan. All DPV, CV, and RDE curves were recorded for decreasing millimolar concentrations of **L** solutions in the electrolyte after adding **L** to the electrolytic cell.

#### 2.1.1. Studies Using the DPV Method

**Figure 4:**
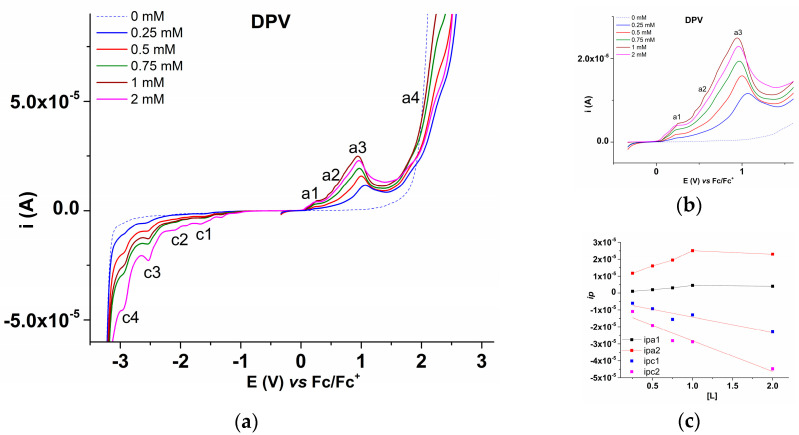
(**a**) DPV curves on glassy carbon for different concentrations of **L** in 0.1 M TBAP/ACN; (**b**) details of a1–a3 anodic domain; (**c**) DPV peak currents vs. concentration of **L** ([**L**]) in 0.1 M TBAP/ACN.

The electrochemical study of **L** started with the DPV method, which was performed at different ligand concentrations ranging from 0 to 2 mM. Several DPV curves are given in Figure 4, which contains the DPV curves recorded in anodic and cathodic scans for different concentrations of **L** (Figure 4a), their detailed anodic domain in the range of a1–a3 processes (Figure 4b), and the plots of anodic (ipa1, ipa2) and cathodic (ipc1, ipc2) peaks vs. **L**’s concentration [**L**] in the supporting electrolyte (Figure 4c).

#### 2.1.2. Studies Using the CV Method

**Figure 5:**
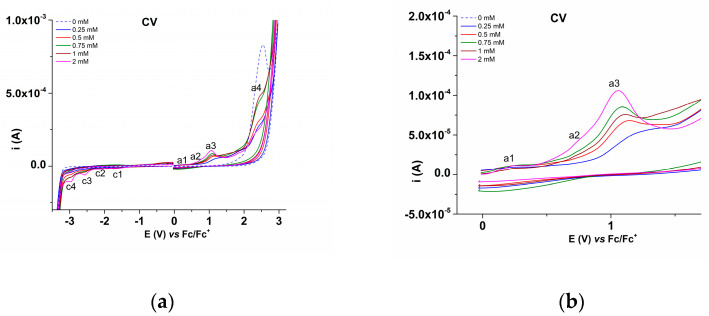
CV curves (0.1 V/s) on glassy carbon for different concentrations of **L** in 0.1 M TBAP/ACN (**a**), and detail of anodic domain (**b**).

**Figure 6:**
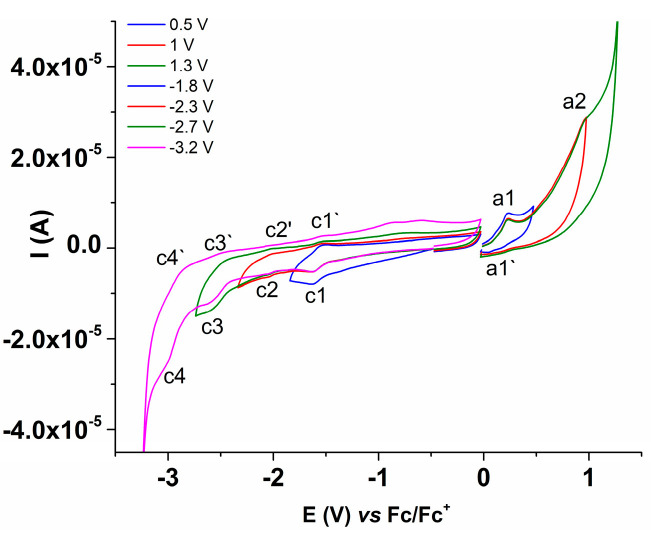
Anodic and cathodic CV curves (0.1 V/s) for **L** (0.17 mM) in 0.07 M TBAP/ACN-DMF (2:1).

**Figure 7:**
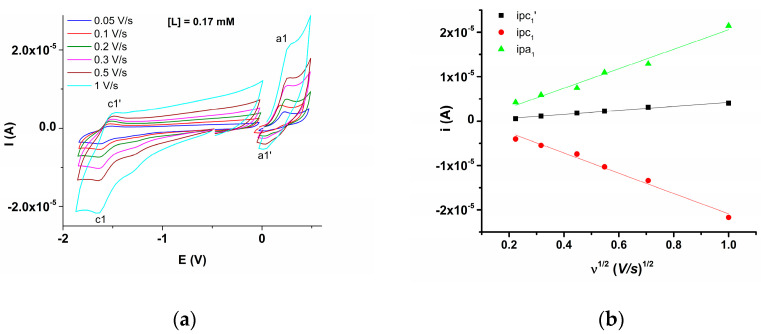
(**a**) CV curves in anodic and cathodic scans at different scan rates for [**L**] = 0.17 mM in 0.07 M TBAP, ACN-DMF (2:1); (**b**) CV peak currents vs. the square root of the scan rate.

The CV study was performed at different concentrations of **L** (0–2 mM). The four anodic peaks and four cathodic peaks, noticed by DPV, were also observed and denoted, respectively, in Figure 5, which contains the CV curves recorded in anodic and cathodic scans for different concentrations of **L** (Figure 5a), and their detailed anodic domain in the range of a1–a3 processes (Figure 5b) in the supporting electrolyte. The CV curves were recorded on variable scan ranges (Figure 6) to highlight the characteristics of the coupled electrochemical processes (which occur during the anodic and cathodic scans on different ranges). For example, the cathodic peak c1 corresponds with the peak c1′ in the return scan, etc. (Figure 6). CV curves were also recorded at different scan rates (Figure 7). The peak currents increase in absolute value linearly with the square root of the scan rate (Figure 7a).

#### 2.1.3. Studies Using the RDE Method

**Figure 8:**
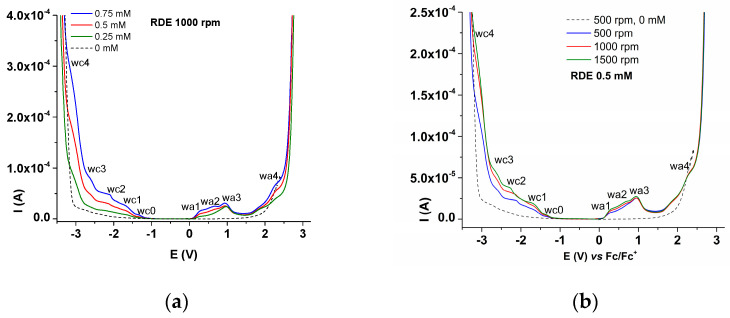
RDE curves at 1000 rpm on glassy carbon at different concentrations of **L** in 0.1 M TBAP/ACN (**a**); RDE curves at different rotation rates on glassy carbon at 0.5 mM concentration of **L** in 0.1 M TBAP/ACN (**b**); all cathodic currents are shown in absolute values.

The RDE study was performed at different concentrations of **L** and different rotation rates of the electrode (Figure 8), which contains the RDE curves recorded in anodic and cathodic scans for different concentrations of **L** (Figure 8a), and the RDE curves at different rotation rates of the electrode in a solution with **L** concentration of 0.5 mM (Figure 8b) in the supporting electrolyte. They evidenced the anodic and cathodic processes, corresponding to the DPV peaks established above, namely wa1–wa4 and wc1–wc4.

### 2.2. Studies of ***L*** by UV-Vis

**Figure 9:**
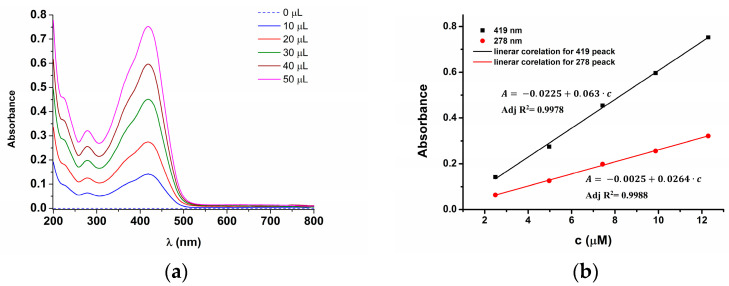
(**a**) UV-Vis spectra obtained for different concentrations of **L** and (**b**) dependence of absorbances on **L** concentration ([**L**]).

UV-Vis studies of the ligand spectra at different concentrations (Figure 9) allowed obtaining UV-Vis characteristics for **L** in ACN and calibration curves at different wavelengths. Figure 9a shows the UV-Vis curves of **L** in ACN solutions, and Figure 9b shows the linear variations in the absorbance at the wavelengths of 419 nm and 278 nm as a function of **L** concentration.

**Figure 10:**
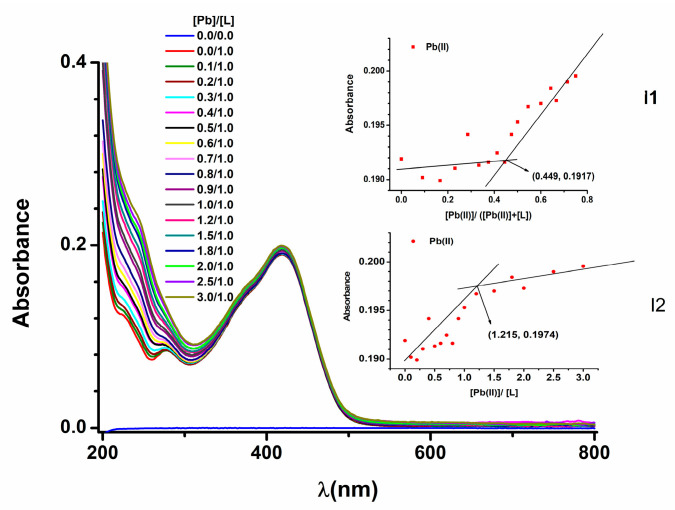
UV-Vis spectra at t = 1 min for different [Pb]/[**L**] ratios; insets: variation in the absorbance for the peak at 419 nm vs. [Pb]/([Pb] + [**L**]) molar fraction (I1 inset) and vs. [Pb]/[**L**] ratio (I2 inset).

The studies on ligand solutions were complemented with UV-Vis studies of the complexation phenomenon of **L** with heavy metal (HM) ions. They were performed on solutions of **L** in ACN in the presence of increasing amounts of HM ions. Figure 10 shows the UV-Vis spectra during the addition of aliquots of Pb(II) to a solution (3.38 µM) of **L** in ACN. The insets I1 and I2 in Figure 10 show the evolution of the absorbance of the peaks at 419 nm vs. [Pb]/[L] ratio and [Pb]/([Pb] + [**L**]) molar fraction, respectively.

Similarly, UV-Vis curves were recorded for the complexation of **L** with ions of Pb, Hg, Cd, and Cu, as well as their evolution with the concentration of each HM ion (Figure S1–Figure S4, respectively)). Figure S1b shows the UV-Vis spectra for the same ratio [HM]/[**L**] = 1 for different HMs: Cd, Pb, Cu, Hg.

### 2.3. DFT Calculation of Quantum Chemical Reactivity Parameters

**Figure 11:**
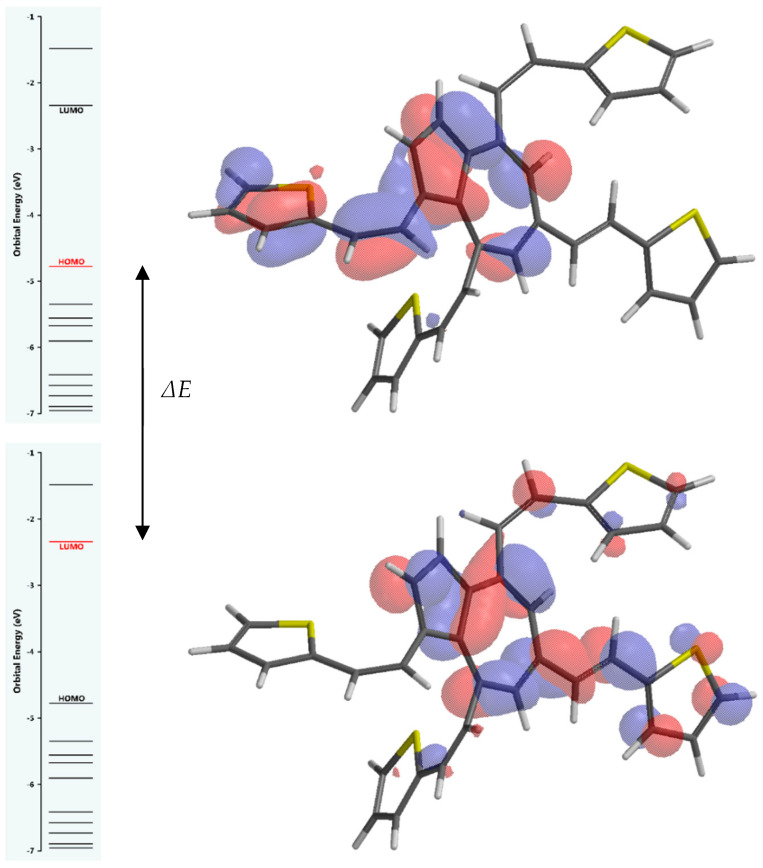
Frontier molecular orbitals energy levels and gap (*ΔE*).

**Figure 12:**
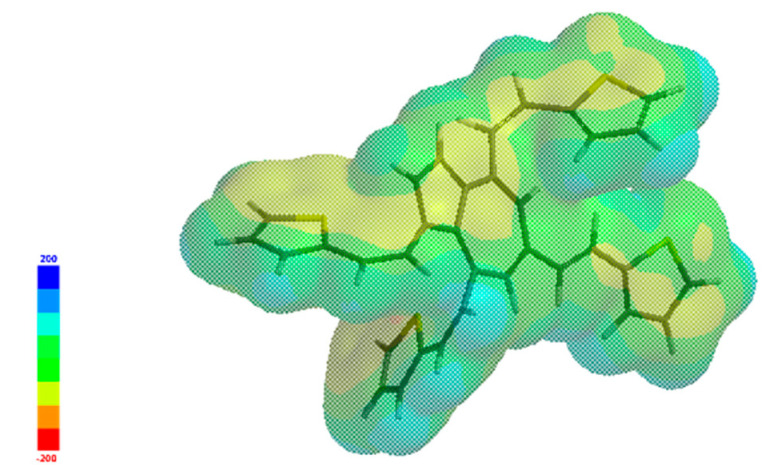
Electrostatic potential map of **L**.

Table 1 reports the computed quantum descriptors associated with the chemical reactivity of **L**: the energy gap (*ΔE*) between HOMO and LUMO energies (*E_HOMO_* − *E_LUMO_*), the ionization potential (*I*), the electron affinity (*A*), the chemical hardness (*η*), the electronegativity (*χ*), the global hardness (*η*), the global softness (*σ*), the chemical potential (*µ*), and the global electrophilicity index (*ω*). Figure 11 illustrates the frontier molecular orbitals’ energy levels and gap, and Figure 12 illustrates the variation in the charge distribution over **L**’ surface, indicating regions of positive (blue) and negative (red-towards orange) electrostatic potential, as well as neutral (green) regions.

### 2.4. Chemically Modified Electrodes Based on ***L***

New chemically modified electrodes based on **L** (**L**-CMEs) were prepared by controlled potential electrolysis (CPE) or by sweeping the potential in the anodic domain from millimolar solutions of **L** ligand in the supporting electrolyte (0.1 M TBAP/ACN) to test the film formation.

**Figure 13:**
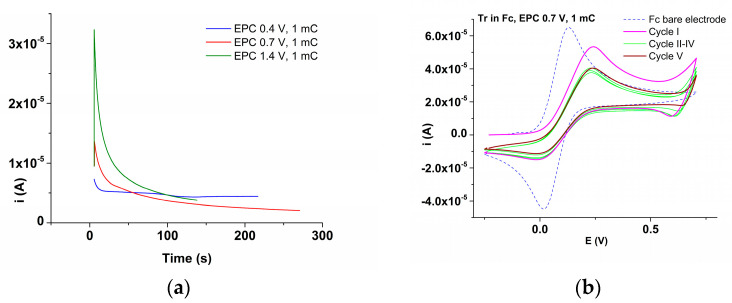
Chronoamperograms during the preparation of **L**-CMEs using an electrical charge of 1 mC in 0.17 mM solution of **L** in 0.1 M TBAP/ACN by CPE at different potentials (**a**); successive CV curves on **L**-CME prepared at 0.7 V and 1mC in ferrocene solution (3 mM), relative to that on the bare (dashed line) electrode (**b**).

The immobilization of **L** was performed on the GC electrode by electropolymerization of **L** at potentials established through the voltammetric measurements in millimolar solutions of **L** in ACN or ACN-DMF. The fixation of the ligand on the freshly cleaned electrode was achieved using chronoamperometry at different anodic potentials and with variable electrical charges. Figure 13a exemplifies some chronoamperograms recorded during the preparation of **L**-CMEs at different applied potentials. After rinsing, the electrodes thus modified were characterized by the redox probe method in ferrocene solutions. An example is given in Figure 13b in which the ferrocene CV curve was recorded before (dashed line) and after the electrode modification in five successive cycles (continuous lines).

**Figure 14:**
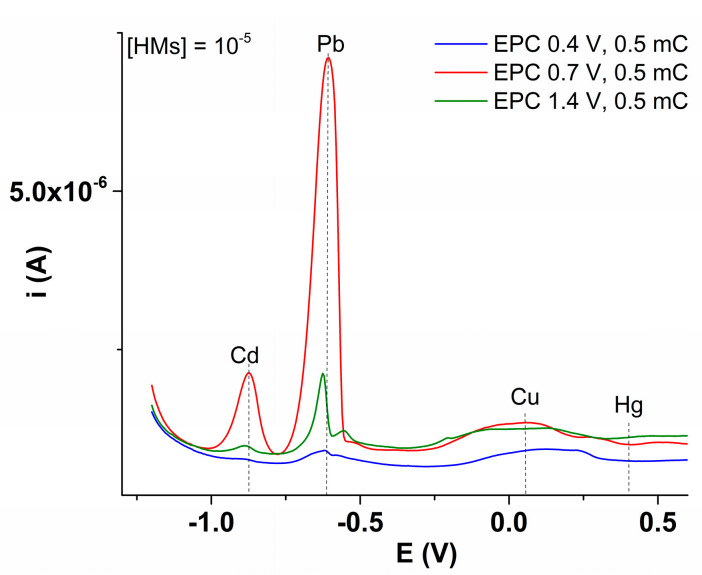
DPV curves recorded on **L**-CMEs prepared by CPE at different potentials and using electropolymerization charges of 0.5 mC after accumulation in aqueous solution of 10^−5^ M concentrations of Cd(II), Pb(II), Cu(II), and Hg(II) ions.

The electrodes modified by CPE were also tested for heavy metal (HM) ion recognition. For this purpose, **L**-CMEs were conditioned as previously shown [25]. The testing of the conditioned electrodes was performed from solutions of equimolar concentrations of HMs, by the anodic stripping method [25], after their polarization at sufficiently negative potentials to ensure the reduction in all HM ions from the complexing film, followed by a slow sweep towards positive potential values, performed using the DPV method. In Figure 14, the DPV curves obtained on **L**-CMEs prepared at different electropolymerization potentials, but using the same electrical charge, are given. All the ions’ signals were labeled. The signals for Pb and Cd were clearly distinguished by their big intensity.

**L**-CMEs were prepared also at different electropolymerization charges at the same potential to see the difference between the formed films. After preparation, they were cleaned by solvent and transferred into a cell containing the supporting electrolyte solution and polarized to see the presence of the film (Figure S6A), and the CV curves were compared with those on bare electrode (see Table S1). Then they were transferred into another cell containing a ferrocene solution and the CV curves were recorded. The ferrocene curves on the modified electrodes obtained in different conditions were compared to those on bare electrodes (Figure S6B). Their characteristics are given in Table S2.

## 3. Discussion

The physicochemical characterization of the ligand (see Supplementary materials) confirmed the structure of 1,4,6,8-tetrakis((E)-2-(thiophen-2-yl)vinyl)azulene (**L**), which was synthesized according to the literature [13]. The electrochemical study of the compound using DPV, CV, and RDE allowed us to highlight the main processes that **L** undergoes at different anodic and cathodic potentials in organic electrolytes. The process currents increase with **L** concentration in all methods (Figure 4, Figure 5 and Figure 8, respectively).

The DPV curves in Figure 4 show the main peaks characteristic of the processes occurring in the anodic (a1–a4) and cathodic (c1–c4) scans. The CV curves in Figure 5 and Figure 6 show that peaks c1, c3, and c4 have response peaks c1′, c3′, and c4′ in the return scans. Since the peak c4′ is weak, it was mentioned as quasi-irreversible in Table 2.

Almost all the processes in the anodic domain are irreversible. Only a1 has a small response (a1′) in the return scan and was mentioned as quasi-irreversible in Table 2. The dependence on concentration of the peak currents is quite linear for the cathodic peaks. For anodic peak a1 and especially for anodic peak a2, it is linearly increasing at small concentrations, and it becomes constant at higher concentrations, in agreement with the formation of films (which are favored by increased concentrations). Due to the complexity of the functional groups and heteroatoms in the molecule of **L**, it is difficult to assign the reactions occurring at each peak.

The CV curves over different potential ranges (Figure 6) and at different scan rates (Figure 7) allowed the estimation of the degree of reversibility of the a1 and c1 processes highlighted during anodic or cathodic scans (Table 2). The variation in the peak current as a function of the square root of the scan rate is linear for peaks c1 and c1′ with variable slopes. The small correlation coefficients reflect complex processes occurring at the anodic potentials (ECE mechanisms).

The RDE curves in Figure 5 obtained at different electrode rotation rates illustrate the high degree of asymmetry between the oxidation and reduction processes. The reduction processes in the cathodic RDE curves (wc1–wc4) appear as classical waves with increasing limiting currents with the concentration and electrode rotation rate. The anodic RDE curves (wa1–wa4) have a classical behavior only for wave wa1, and for waves wa2 and wa3, the behavior is consistent with the formation of films at these potentials (they appear as peaks). With a further increase in potential, the currents decrease, reaching values close to those for the supporting electrolyte. The wa4 process appears as a regular process which, however, takes place in parallel with the discharge of the supporting electrolyte.

**Figure 15:**
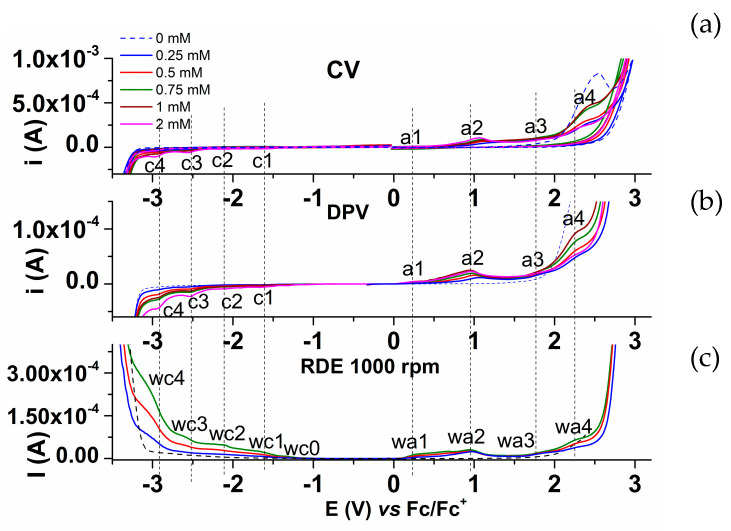
CV (**a**), DPV (**b**), and RDE (**c**) curves on a glassy carbon electrode for different concentrations of the ligand in 0.1 M TBAP, ACN.

Figure 15 brings together the curves obtained for **L** at different concentrations by CV, DPV, and RDE methods, which presented in parallel, show a good agreement of the processes highlighted at different potentials. The lower reversibility of the a1 peak, expressed by CV (compared to the c1 peak corresponding to the first electron transfer in the cathodic scan, as seen in Figure 6) shows that the ligand **L** can undergo irreversible electropolymerization processes at anodic potentials, leading to modified electrodes covered with insulating films. Cathodic processes take place in solution on the electrode surface not covered with films. The study of the surfaces of the modified electrodes, obtained at anodic potentials applied by controlled potential electrolysis (CPE), carried out using SEM, XPS, and Raman methods, confirmed the formation of films by electropolymerization of this ligand. The shape of the anodic DPV, CV, and RDE curves is consistent with the formation of films by electrooxidation at potentials starting from 0.4 V, as will be discussed below.

Thiophenylvinylazulene **L** possesses several UV-Vis bands. Two bands are important for determining the color: the major visible band, representing the S2-S0 transition between the LUMO+1 and HOMO orbitals, allowed by the orbital geometries, and the second band, representing the S1-S0 transition, between LUMO and HOMO, forbidden by the orbital geometry [13]. The last band is very weak and is divided into a multitude of mini-bands that influence the color of compound **L**. Our studies performed in acetonitrile (ACN) revealed a spectral shape like that obtained in methanol [13], but with a Vis absorption maximum at 419 nm. By recording **L** spectra in the presence of different amounts of ligand (Figure 9a), a linear increase in peak intensity with [**L**] was observed. From the representation of peak heights vs. **L** amount (Figure 9b), the extinction coefficients were calculated at two transition peaks in visible and UV domains (Table 3). Their values were compared with those obtained in methanol [13]. Analysis of basic UV-Vis properties of **L,** given in Table 3, showed that **L** presents a small positive solvatochromism. The visible band in methanol (with dipole moment of 5.1 D) is shifted toward higher wavelengths in acetonitrile (with dipole moment of 5.8 D). The increase in solvent dipole moment leads to the extinction coefficient increase.

The studies of UV-Vis spectra performed on solutions of **L** in ACN in the presence of increasing amounts of HM ions evidence the complexation phenomenon of **L** with heavy metal (HM) ions. In Figure 10, we can see the evolution of UV-Vis spectra during the addition of Pb(II) to **L** in ACN. Insets I1 and I2 in Figure 10 show that the absorbance of the peak at 419 nm decreases linearly versus [Pb]/([Pb] + [**L**]) molar fraction and [Pb]/[**L**] molar ratio, respectively. The application of the molar ratio method [33,34] and of the Molland method [35] led to concordant values of the ratio between the concentrations of [Pb(II)] and [**L**] in their complex. This can be evaluated at 0.5, which corresponds to the molecular formula PbL_2_.

The UV-Vis spectra evolution for various number of equivalents of Pb ([Pb]/[**L**] ratio of 0, 1, 2, 3) can be followed in Figure S1a. It affects especially the UV domain, but there is also a small change in the visible band. From Figure S1b, where the spectra of solutions of investigated HMs (Cd, Pb, Hg, Cu) at 1:1 ratio in respect to **L** were compared, the four ions present two types of behavior. The curves for Hg and Pb overlap, as do those for Cu and Cd.

This behavior is confirmed in the study of the complexation of each ion, presented in Figures S2a, S3a and S4a for variable complexation ratios and in Figures S2b, S3b and S4b for integer numbers of equivalents (between 0 and 3) of Hg, Cd, and Cu ions, respectively. These results show that the homogeneous complexation of ligand **L** with Pb and Hg ions is different from that with Cu and Cd ions.

The results of DFT calculations for **L**, presented in Table 1, show that the computed quantum descriptors are associated with the chemical reactivity of **L**. The calculated total energy difference between HOMO and LUMO allows the estimation of the band gap related to optical properties and the kinetic stability of the ligand. The value of the band gap of 2.44 eV indicates a moderate level of stability and reactivity of **L**. Generally, a smaller gap (around 2 eV or less) suggests higher reactivity and lower stability, while larger gaps (4 eV or more) indicate lower reactivity and higher stability [36]. According to the calculated value of 2.44 eV, as illustrated in Figure 11, it could be considered that **L** is neither extremely reactive nor exceptionally stable, falling within a range where it might be useful for particular applications such as organic solar cells or optoelectronics.

From Figure 12, which illustrates the variation in the charge distribution over **L**’ surface, a higher electron density is noticed over the sulfur atoms of the thiophene rings. The presence of four electron-rich heterocycles as thiophene in the structure of **L** influences the overall molecule’s reactivity and intermolecular interactions. It can lead to stronger interactions with electrophiles (such as HM ions) or other molecules.

The use of the films formed by ligand electropolymerization in analytical experiments confirms the heavy metal ion recognition. Chemically modified electrodes (CMEs) prepared at various potentials of **L** by electrooxidation of **L** show a maximum response intensity for Pb compared to the other cations, as seen in Figure 14. They were characterized by electrochemistry and were tested for the analysis of heavy metal (HM) ions.

The immobilization of **L** on GC electrode leads to chemically modified electrodes based on **L** (**L**-CMEs) with variable properties according to preparation conditions. The electropolymerization of **L** at variable potentials lead to big differences in their properties from an analytical point of view, as seen in Figure 14. Also, the applied current leads to changes in their behavior, investigated in the solutions of supporting electrolyte (Figure S6a) and in ferrocene solutions (Figure S6b). The examination of Figure S6a shows us the presence of a characteristic signal for the film which appears on the modified electrode in the supporting electrolyte solution in the absence of ligand in solution, at potentials appropriate to those for ligand oxidation. The evolution of its characteristics can be followed in Table S3. Its peak potential is around +0.82 V, and it is shifted toward more positive values when its thickness increases (for films formed using higher electrical charges). Its current initially increases from 0.5 mC to 1 mC but is constant or slowly decreases at higher electrical charges used in CPE. This indicates the formation of compact films. Also, from Figure 13b, it is observed that the ferrocene signal is displaced from that on the unmodified electrode (dashed line). It continues to change (Figure 13b) on the modified electrode in five successive cycles (continuous lines), which proves the continuation of polymerization by successive cycling. Particularly interesting are the films formed at low anodic potentials (not presented in the paper) which appear conductive (the ferrocene signal increases on the modified electrode).

The obtained *CMEs* were also tested for the analysis of synthetic samples of heavy metal (HM) ions—Cd(II), Pb(II), Cu(II), and Hg(II)—by anodic stripping. They were found to be specific for Pb(II) and Cd(II) ions (Figure 14). The preparation conditions have influence on the electrochemical response (Figure 14 and Figure S6, Tables S2 and S3).

Optimization of the preparation conditions of CMEs to find a targeted analytical response is ongoing and will be the subject of specific studies, which aim to correlate the surface properties of the modified electrodes with the electrocatalytic activity [37] and with the analytical response of the modified electrodes based on this ligand.

## 4. Materials and Methods

**Scheme 1:**
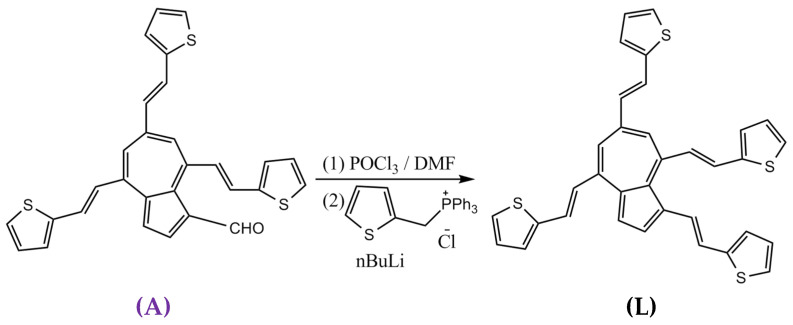
Synthesis of the ligand 1,4,6,8-tetrakis((E)-2-(thiophen-2-yl)vinyl)azulene (**L**).

The ligand 1,4,6,8-tetrakis((E)-2-(thiophen-2-yl)vinyl)azulene (**L**) was synthesized by the sequence Vilsmeier–Wittig reactions (Scheme 1) in good yields (step 1, yield 63%) + (step 2, yield 55%), starting from 4,6,8-tris((E)-2-(thiophen-2-yl)vinyl)azulene-1-carbaldehyde (**A**), phosphoryl chloride (POCl_3_), and triphenyl(thiophen-2-ylmethyl) phosphonium chloride, according to the previously published procedure [13]. The main characteristics of L (characterization by elemental analysis, UV-Vis, 1H NMR, 13C-NMR, IR, and MS) are given in the Supplementary Material.

Acetonitrile (ACN, electronic grade 99.999% trace metals) and dimethyl formamide (DMF) with low water content were from Sigma Aldrich (Steinheim, Germany) and used as solvents for the optical and electrochemical experiments. Tetrabutylammonium perchlorate (TBAP, Fluka, Munich, Germany, analytical purity ≥ 99.0%) was used as a supporting electrolyte.

For heavy metal analysis, we used the following heavy metal salts: mercury (II) acetate (Fluka, Munich, Germany ≥ 98%), cadmium nitrate tetrahydrate (Fluka, Munich, Germany ≥ 98%), copper (II) acetate monohydrate (Fluka, Munich, Germany, ≥98%), and lead (II) nitrate (Fluka, Munich, Germany ≥ 99.5%).

Electrochemical experiments were performed on Autolab PGSTAT302N (Utrecht, Nederlands) potentiostat connected to three-electrode electrochemical cells. For electrochemical characterization and preparation of CMEs, the working electrode (WE) was a glassy carbon (GC) disk (Metrohm, Herisau, Switzerland) with a diameter of 3 mm, the counter electrode (CE) was a platinum wire, and the reference electrode (RE) was Ag/10 mM Ag-NO_3_, 0.1 M TBAP/CH_3_CN. All potential values were reported to the ferrocene/ferrocenium redox couple potential (Fc/Fc^+^) at the end of each experiment. For the electrochemical analysis of HM ions in aqueous solutions using CMEs, a three-electrode cell (transfer cell) was used, in which the WEs were GC disks modified with **L** films, the reference electrode was Ag/AgCl, 3 M KCl, and the auxiliary electrode was a platinum wire. Electrochemical experiments for the characterization of **L** were carried out in a controlled argon atmosphere.

The electrooxidation and electroreduction curves were recorded on a glassy carbon (GC) electrode in millimolar solutions of **L** in organic solvents such as ACN or mixtures 2:1 (in volumes) of ACN and DMF (ACN-DMF) using tetrabutylammonium perchlorate (TBAP) as a supporting electrolyte. The background was initially recorded separately on the freshly cleaned GC electrode in the supporting electrolyte. **L** was then added to the electrochemical cell, and the curves were recorded for **L** solutions in anodic or/and cathodic scan. Then, the electrode was polished again on electrochemical velvet with diamond paste, cleaned with the solvent, and dried. Cyclic voltammetry (CV), differential pulse voltammetry (DPV), and rotating disk electrode voltammetry (RDE) methods were used for testing various solutions of **L** with millimolar concentrations, and in different conditions according to the applied method. The CV curves were recorded at scan rates between 0.05 and 0.5 V/s. DPV curves were obtained with a pulse height of 0.025 V and a time step of 0.2 s at 0.01 V/s. RDE curves were recorded with the electrode rotation rates from 500 to 1500 rpm at 0.01 V/s.

The chemically modified electrodes based on **L** (**L**-CME) were prepared from millimolar solutions of **L** in 0.1 M TBAP in ACN or DMF by potential sweep or controlled potential electrolysis (CPE). The GC electrode was inserted into the preparation cell containing L solution in the supporting electrolyte and was biased to a defined potential which was maintained until a defined electrical charge was reached. The resulting CME was removed from the preparation solution, blotted with fine paper, and rinsed with CH_3_CN. It was then introduced and conditioned in the transfer cell (containing 0.1 M acetate buffer solution, pH = 4.5), equilibrated (15 CV cycles at 0.1 V/s between −0.89 V and 0.6 V), and superoxidized (15 CV cycles at 0.1 V/s between -0.19 V and 1.85 V). Then, the conditioned CME was introduced into the HM mixture containing 5.10^−5^ M of each cation (Cd^2+^, Pb^2+^, Cu^2+^, Pb^2+^, Cu^2+^, and Hg^2+^) in deionized water under controlled stirring. After 15 min, the electrode was removed and placed in 0.1 M acetate buffer solution (pH = 4.5) and held for 3 min at -1 V; then, a DPV scan (0.01 V/s) from −1 V to 0.6 V was initiated. The resulting DPV curve was recorded, and the current for each peak was measured relative to baseline.

The UV-Vis studies were performed on a JASCO V-670 (Tokyo, Japan) spectrometer in 1 cm optical path quartz cuvettes. For the ligand study, the spectra of different concentrations of ligand in acetonitrile were recorded vs. acetonitrile. For the study of HM ion complexation, the HM ions were added from the stock solutions as aliquots in demineralized water in the cuvette containing the ligand solved in acetonitrile under stirring. The spectra recorded after 1, 5, 10, and 15 min were found to be stable, and the spectra after **1** min were finally kept and compared. The determination of the complexation ratio was performed using the method of continuous variations (Job) [33,34] and Molland method [35].

All experiments were conducted at 25 °C.

DFT investigation was conducted using the B3LYP/6-31(d,p) method to determine reactivity-related properties for **L** at the equilibrium geometry, in the ground state. For property computations, we used Spartan Software 14 (Wavefunction Inc., Irvine, CA, USA) using B3LYP/6-31(d,p) methodology, like in our previous studies on different azulene derivative structures [25,32].

## 5. Conclusions

Comprehensive electrochemical investigations of 1,4,6,8-tetrakis((E)-2-(thiophen-2-yl)vinyl)azulene (**L**) demonstrated its pronounced electropolymerization capacity, which was experimentally confirmed through the successful preparation of chemically modified electrodes (CMEs). These CMEs exhibited selective recognition properties toward Pb(II) and Cd(II), with their analytical responses strongly influenced by the electrode preparation conditions. In contrast, UV-Vis optical studies revealed a different complexation order, indicating selective homogeneous complexation with Pb(II) and Hg(II) ions.

Theoretical insights obtained by DFT calculations were consistent with the experimental electrochemical and spectroscopic findings, confirming the moderate reactivity of **L** and highlighting the role of its conjugated thiophen-2-yl)vinyl-azulene framework in dictating redox and binding behavior. This correlation between theory and experiment provides a robust foundation for understanding the structure–function relationships of such ligands.

Taken together, these results establish **L** as a promising functional material for electrode surface modification and as a versatile platform for the development of advanced electrochemical sensors. Its combined optical, electrochemical, and theoretical properties recommend it for further exploration in heavy metal ion detection and potential optoelectronic applications.

## Figures and Tables

**Table 1:** Calculated quantum reactivity parameters according to Koopmans’ theorem.

Quantum Chemical Reactivity Parameter Symbol (Unit)	Formula	Value
FMOs energy gap, *ΔE* (eV)	*E*_HOMO_ − *E*_LUMO_	2.44
Ionization potential, *I* (eV)	*I* = − *E*_HOMO_	4.78
Electron affinity, *A* (eV)	*A* = − *E*_LUMO_	2.34
Electronegativity, *χ* (eV)	*χ* = (*I* + *A*)/2	3.56
Global hardness, *η* (eV)	*η* = (*I* − *A*)/2	1.22
Global softness, *σ* (eV^−1^)	*σ* = 1/*η*	0.82
Chemical potential, *µ* (eV)	*µ* = (*E*_HOMO_ + *E*_LUMO_)/2	−3.56
Global electrophilicity index, *ω* (eV)	*ω* = *µ*^2^/2 *η*	5.19

**Table 2:** Peak potentials (V) and electrochemical characteristics of CV and DPV peaks noticed on GC electrode in **L** solution in 0.1 M TBAP/ACN during anodic and cathodic scans.

Peak	Method		Characteristics of the Peak
	DPV	CV	
a1	0.25	0.30	Quasireversible
a2	0.72	0.85	Irreversible
a3	0.96	1.10	Irreversible
a4	2.27	2.40	Irreversible
c1	−1.60	−1.70	Reversible
c2	−2.16	−2.20	Irreversible
c3	−2.52	−2.60	Reversible
c4	−2.94	−3.01	Quasireversible

**Table 3:** Extinction coefficient (ε) and maximum wavelength (λ) values in ACN (ε, λ) and methanol (ε’, λ’) for **L** solutions.

λ (nm)	ε (M^−1^cm^−1^)	log(ε)	λ’ (nm)	log(ε’)
278	26,400	4.42	279	4.14
419	63,400	4.80	418	4.58

## Data Availability

Raw experimental and computed data supporting the results of this study are available upon reasonable request.

## Supplementary Materials

The following supporting information can be downloaded at: https://www.mdpi.com/article/10.3390/molecules30183762/s1, Figure S1: UV-Vis spectra at t = 1 min for various [Pb]/[**L**] ratios of 0, 1, 2, 3 (a) and for [HM]/[**L**] = 1 for Cd, Pb, Hg, Cu (b); Figure S2: UV-Vis spectra at t = 1 min for various [Hg]/[**L**] ratios (a) and for the ratios [Hg]/[**L**] of 0, 1, 2, 3 (b); Figure S3: UV-Vis spectra at t = 1 min for various [Cd]/[**L**] ratios (a) and for the ratios [Cd]/[**L**] of 0, 1, 2, 3 (b); Figure S4: UV-Vis spectra at t = 1 min for various [Cu]/[**L**] ratios (a) and for the ratios [Cu]/[**L**] of 0, 1, 2, 3 (b); Figure S5: Atom labeling of **L** given by Spartan software; Figure S6: CV curves recorded on **L**-CMEs prepared by CPE at 0.7 V and using different electropolymerization charges (0.5 mC–5 mC) in 0.1 M TBAP/DMF (a) and in 3 mM solution of ferrocene in 0.1 M TBAP/DMF (b);. Table S1: Cartesian coordinates (Å) of **L**’ structure; Table S2: Ferrocene couple characteristics on **L**-CMEs prepared on GC electrode (3 mm in diameter) by CPE at 0.7 V and using different electropolymerization charges: anodic peak potential (Epa) and current (ipa), cathodic peak potential (Epc) and current (ipc), difference between Epa and Epc (ΔEp), formal potential (Ef); Table S3: Characteristics of the process appeared in the CV curves recorded in 0. M TBAP/DMF supporting electrolyte on **L**-CMEs, after their preparation by CPE at 0.7 V and using different amounts of electrical charge, vs. those recorded on GC bare electrode (dashed line in Figure S6): peak potential (Epa) and current (ipa).
