# Supplementary material for: Electrochemical and Optical Experiments and DFT Calculations of 1,4,6,8-Tetrakis((E)-2-(thiophen-2-yl)vinyl)azulene
Source: Molecules. 2025 Sep 16;30(18):3762. doi: 10.3390/molecules30183762 (PMC12473093; doi:10.3390/molecules30183762)
Supplement: Supplementary file 1 [file molecules-30-03762-s001.zip › molecules-3867258-supplementary.pdf]

Supplementary Materials

Electrochemical and optical experiments and DFT calculations of 1,4,6,8-tetrakis((*E*)-2-(thiophen-2-yl)vinyl)

Cornelia Musina (Borsaru)[1], Alina-Giorgiana Brotea[1,2], Mihaela Cristea[3], Gabriela Stanciu[2*], Amalia Stefaniu[4], Eleonora-Mihaela Ungureanu[5*]

Basic properties for **L2064** and characterization by elemental analysis, UV-Vis, ^{1}H NMR, ^{13}C-NMR, IR, MS:

1,4,6,8-tetrakis((E)-2-(thiophen-2-yl)vinyl)azulene, **L**, Dark green crytals, m.p. 227 ^{o}C. UV-Vis, λ_{max} (lg ε): 201 (4.36), 221 (4.25), 279 (4.14), 418 (4.58) nm. ^{1}H-NMR, δ (ppm): 6.92 (dd, ^{3}J = 5.0 Hz, ^{4}J = 3.6 Hz, 1 H, 4-H'), 6.97 (d, ^{3}J = 3.6 Hz, 1 H, 3-H'), 7.06 (d, ^{3}J = 16.2 Hz, 1 H, CH$^{\beta'}$), 7.07 (d, ^{3}J = 5.2 Hz, 1 H, 4-H''), 7.40 (d, ^{3}J = 16.2 Hz, 1 H CH$^{\beta'}$), 7.02 (dd, ^{3}J = 5.0 Hz, ^{4}J = 3.6 Hz, 1 H, 4-H'''), 7.04 (dd, ^{3}J = 5.0 Hz, ^{4}J = 3.6 Hz, 2 H, 4-H'', 4-H^{iv}), 7.08 (d, ^{3}J = 16.2 Hz, 1 H, CH$^{\beta'''}$), 7.20 (d, ^{3}J = 3.6 Hz, 1 H, 3-H'''), 7.22 (d, ^{3}J = 3.6 Hz, 1 H, 3-H''), 7.24 (d, ^{3}J = 3.6 Hz, 1 H, 3-H^{iv}), 7.27 (d, ^{3}J = 5.2 Hz, 1 H, 5-H '''), 7.29 (d, ^{3}J = 5.2 Hz, 1 H, 5-H''), 7.31 (d, ^{3}J = 5.0 Hz, 1 H, 5-H^{iv}), 7.69 (d, ^{3}J = 15.9 Hz, 1 H CH$^{\alpha'''}$), 7.72 (d, ^{3}J = 15.9 Hz, 1 H CH$^{\alpha''}$), 7.76 (d, ^{3}J = 15.9 Hz, 2 H CH$^{\alpha iv}$), 7.48 (d, ^{3}J = 4.0 Hz, 1 H, 3- H), 7.42 (s, 1 H, 5-H), 7.43 (s, 1 H, 7-H), 7.88 (t, ^{3}J = 4.0 Hz, 1 H, 2-H). ^{13}C-NMR, δ (ppm): 116.0, 116.8, 119.9, 121.4, 121.7, 123.1, 123.3, 123.7, 124.3, 124.6, 124.7, 125.2, 125.6, 125.7, 125.8, 125.9, 126.5, 126.6, 127.1, 127.3, 127.6, 127.7, 127.9, 128.4, 128.5, 128.6, 128.7, 129.6, 130.3, 130.6, 133.0, 133.4, 133.6, 133.8, 134.0, 139.0, 140.2, 142.3, 142.5, 142.6, 142.9, 142.9, 143.9, 144.4, 145.1. MS [ESI]: 561 [M+1]. Calcd. for $C_{34}H_{24}S_4$: C, 72.82; H, 4.31; S, 22.87. Found: C, 72.81; H, 4.32; S, 22.87.

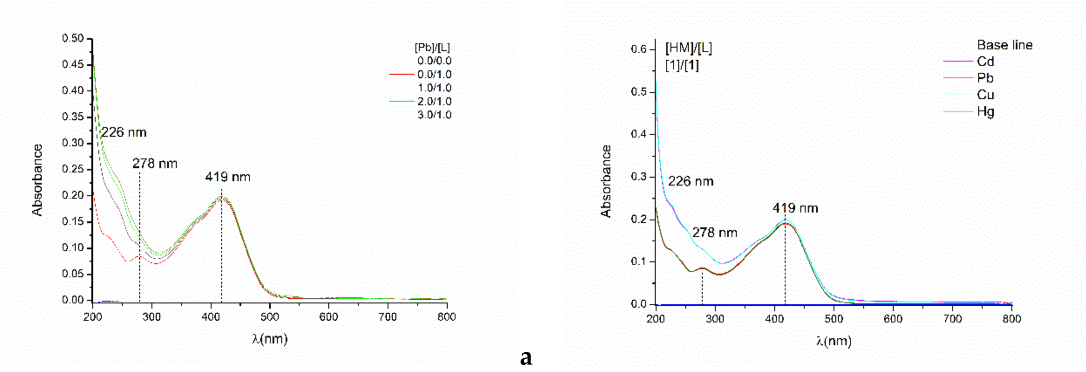

Figure S1. UV-Vis spectra at t = 1 min for various [Pb]/[**L**] ratio of 0, 1, 2, 3 (a) and for [HM]/[**L**] = 1 for Cd, Pb, Hg, Cu. (b).

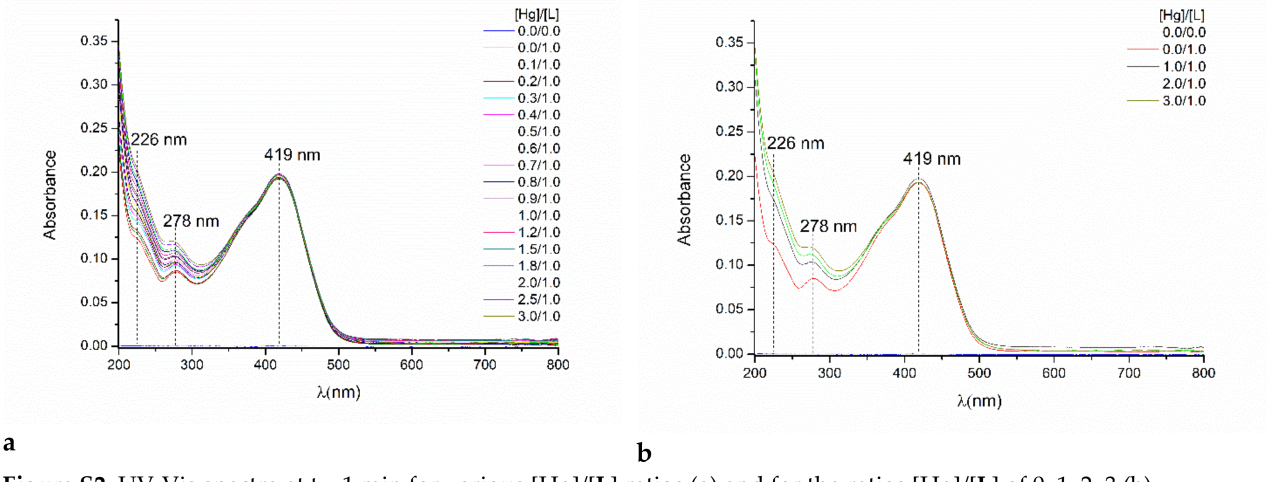

Figure S2. UV-Vis spectra at t = 1 min for various [Hg]/[**L**] ratios (a) and for the ratios [Hg]/[**L**] of 0, 1, 2, 3 (b).

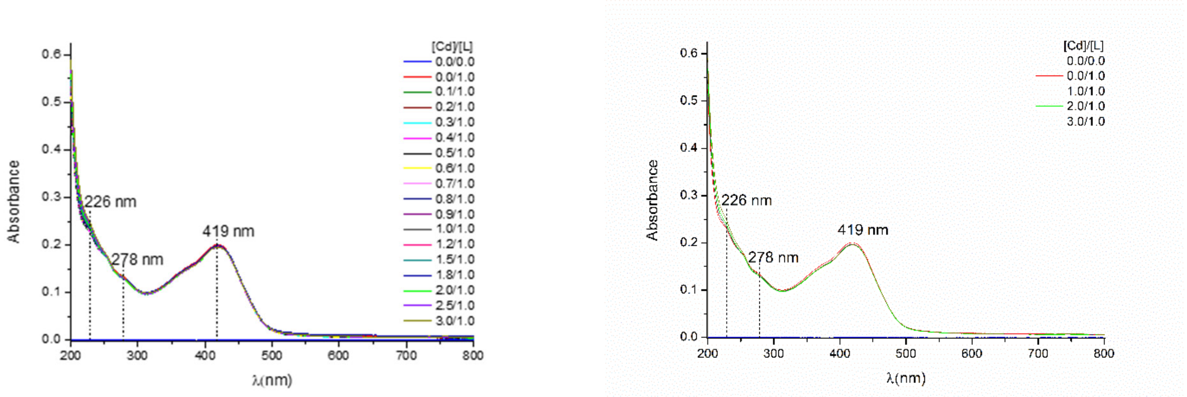

a **b**

Figure S3. UV-Vis spectra at t = 1 min for various [Cd]/[**L**] ratios (a) and for the ratios [Cd]/[**L**] of 0, 1, 2, 3 (b).

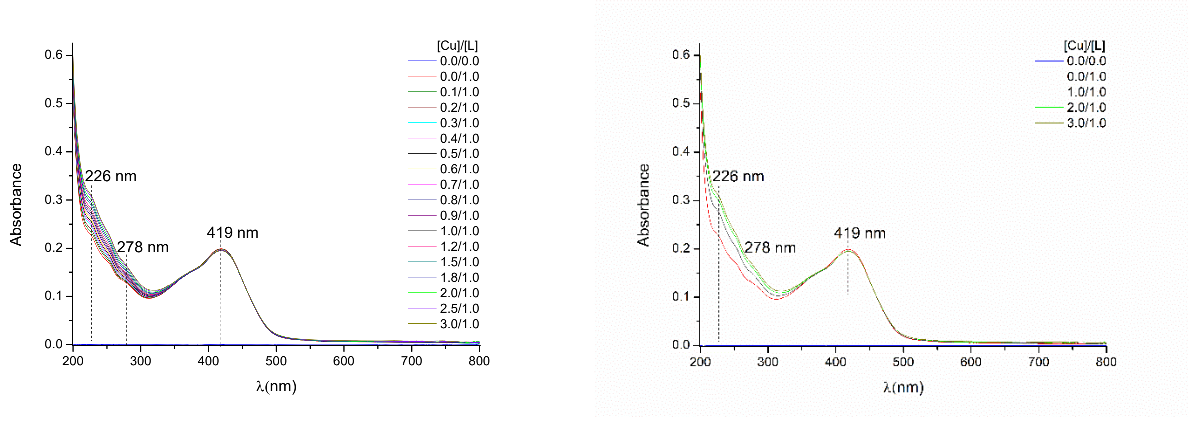

a **b**

Figure S4. UV-Vis spectra at t = 1 min for various [Cu]/[**L**] ratios (a) and for the ratios [Cu]/[**L**] of 0, 1, 2, 3 (b).

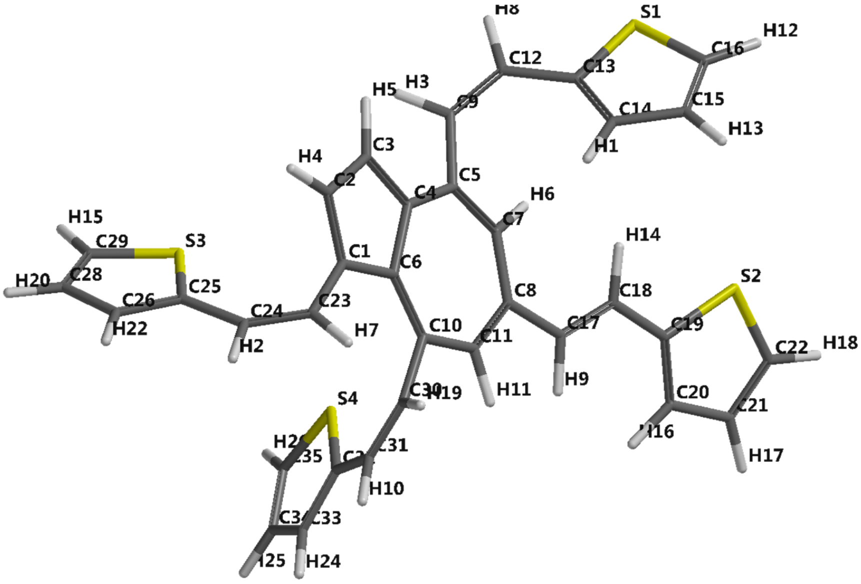

Figure S5. Atom labelling of **L** given by Spartan software.

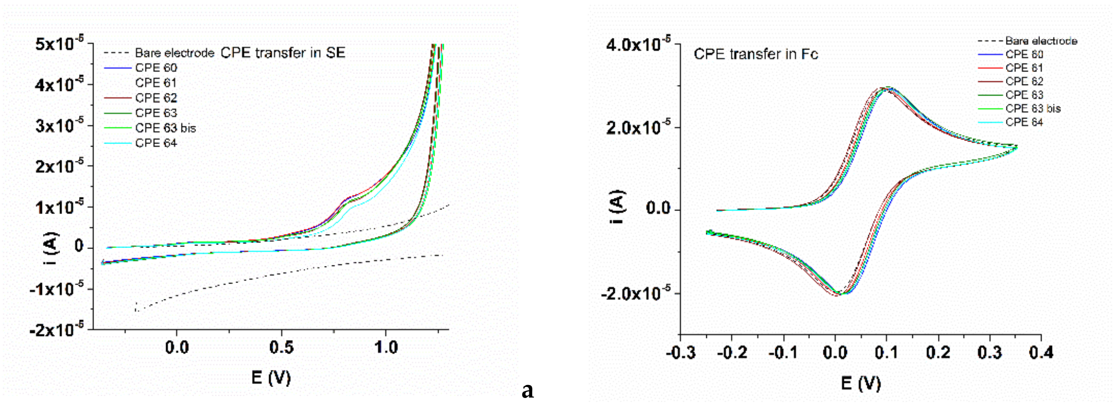

Figure S6. CV curves recorded on **L**-CMEs prepared by CPE at 0.7 V and using different electropolymerization charges (0.5 mC - 5 mC) in 0.1 M TBAP in ACN (**a**) and in 3 mM solution of ferrocene in 0.1 M TBAP in ACN (**b**).

Table S1. Cartesian coordinates (Å) of L′structure.

Atom	X	Y	Z
C1	2.7259523	1.2818000	-1.0994045
C2	2.7448332	2.5718335	-0.5249125
H4	3.5883148	3.2490985	-0.5938644
C3	1.5298150	2.8476372	0.1033850
H5	1.2716067	3.7746343	0.5970311
C4	0.6821369	1.7367394	-0.0449185
C6	1.4427953	0.7171712	-0.8391433
C5	-0.6165344	1.6561215	0.5008012
C7	-1.5231608	0.5984999	0.3840009
H6	-2.4465050	0.7444794	0.9325382
C8	-1.4160493	-0.6252937	-0.2999022
C11	-0.2783634	-1.1178048	-0.9703024
C10	0.9879142	-0.5632032	-1.2123397
H11	-0.4081289	-2.1093205	-1.3962556
C9	-1.0434161	2.7826670	1.3714725
H3	-0.3172191	3.0707009	2.1289668
C12	-2.2020963	3.4767873	1.3631539
H8	-2.3221742	4.2085899	2.1612340
C13	-3.3112186	3.4270028	0.4187278
C14	-3.3850802	2.9846909	-0.8866459
H1	-2.5355708	2.5660971	-1.4120065
C15	-4.6688673	3.1552954	-1.4750364
H13	-4.9064848	2.8651224	-2.4928695
C16	-5.5749159	3.7339256	-0.6256225
H12	-6.6132228	3.9718613	-0.8137242
S1	-4.8660828	4.0834952	0.9135809
C17	-2.5785701	-1.5221334	-0.3263684
H9	-2.3616528	-2.5501926	-0.6106018
C18	-3.8674205	-1.1811455	-0.0904779
H14	-4.1099808	-0.1427795	0.1241394
C19	-4.9926943	-2.0920417	-0.1337856
C20	-5.0395361	-3.4645315	-0.2907824
H16	-4.1503582	-4.0769417	-0.3934868
C21	-6.3578166	-3.9922035	-0.2913834
H17	-6.5836641	-5.0474875	-0.4014095

C22	-7.3204430	-3.0285872	-0.1318753
H18	-8.3940686	-3.1541221	-0.0929557
S2	-6.6219323	-1.4534763	0.0214923
C23	3.8427535	0.7540801	-1.8941643
H7	3.6204636	0.4659622	-2.9195856
C24	5.1434525	0.6611115	-1.5357262
H2	5.8371973	0.3476687	-2.3159116
C25	5.8076582	0.8446084	-0.2576821
C26	7.1740486	0.7565395	-0.0682362
C28	7.5833825	0.8851028	1.2871033
H20	8.6170056	0.8441048	1.6140553
C29	6.5261362	1.0641652	2.1368761
H15	6.5441994	1.1931129	3.2111564
S3	5.0113087	1.0893394	1.2910004
C30	1.8785328	-1.4441109	-2.0242749
H19	2.0688320	-1.1081318	-3.0409430
C31	2.4225135	-2.6237108	-1.6652039
H10	2.9992522	-3.1413173	-2.4315535
C32	2.4312718	-3.3359721	-0.3998588
C33	3.0636028	-4.5477811	-0.1971384
H24	3.5841461	-5.0666818	-0.9959222
C34	2.9769542	-5.0306125	1.1363999
H25	3.4146851	-5.9643644	1.4723670
C35	2.2806828	-4.1815769	1.9539523
H26	2.0608522	-4.2962441	3.0072714
S4	1.7134723	-2.7815676	1.1044299
H22	7.8602267	0.5940314	-0.8936002

Table S2. Ferrocene couple characteristics on L-CMEs prepared on GC electrode (3 mm in diameter) by CPE at 0.7 V and using different electropolymerization charges: anodic peak potential (Epa) and current (ipa), cathodic peak potential (Epc) and current (ipc), difference between Epa and Epc (ΔEp), formal potential (Ef);

Crt. Nr.	Charge (mC) used in CPE (CME's name)	Epa (V)	$10^5 \cdot$ ipa (A)	Epc (V)	$10^5 \cdot$ ipc (A)	ΔEp (V)	Ef (V)
1	Bare electrode	0.091	2.906	0.002	−1.979	0.089	0.465
2	0.5 mC (CME 60)	0.103	2.930	0.021	−2.028	0.082	0.620
3	1 mC (CME 61)	0.093	2.930	0.012	−2.028	0.081	0.525
4	2 mC (CME 62)	0.089	2.954	0.002	−2.062	0.087	0.455
5	3 mC (CME 63)	0.102	2.986	0.012	−2.005	0.090	0.570
6	3 mC (CME 63bis)	0.103	2.920	0.012	−2.010	0.091	0.575
7	5 mC (CME 64)	0.102	2.922	0.012	−2.031	0.090	0.570

Table S3. Characteristics of the process appeared in the CV curves recorded in 0. M TBAP/ACN supporting electrolyte on **L**-CMEs, after their preparation by CPE at 0.7 V and using different amounts of electrical charge, *vs.* those recorded on GC bare electrode (dashed line in Figure S6): peak potential (Epa) and current (ipa).

Crt. Nr.	E_{CPE} (V)	Epa (V)	$10^5 \cdot$ ipa (A)
1	Bare electrode	-	0.397
2	0.5 mC (CME 60)	0.820	1.210
3	1 mC (CME 61)	0.820	1.241
4	2 mC (CME 62)	0.813	1.104
5	3 mC (CME 63)	0.831	1.167
6	3 mC (CME 63bis)	0.831	1.167
7	5 mC (CME 64)	0.845	1.019
